# A Prospective, Randomized Trial of Two Mucin Secretogogues for the Treatment of Dry Eye Syndrome in Office Workers

**DOI:** 10.1038/s41598-017-13121-9

**Published:** 2017-11-09

**Authors:** Jun Shimazaki, Den Seika, Masamichi Saga, Kazumi Fukagawa, Miki Sakata, Miki Iwasaki, Takashi Okano

**Affiliations:** 1 Department of Ophthalmology, Tokyo Dental College Ichikawa General Hospital, Chiba, Japan; 2 Shimazaki Eye Clinic, Tokyo, Japan; 3 Ichikawa Shapo Eye Clinic, Chiba, Japan; 4 Ryogoku Eye Clinic, Tokyo, Japan; 5 Shinjuku Eye Clinic, Tokyo, Japan; 6 Iidabashi Eye Clinic, Tokyo, Japan; 7 Smile Eye Clinic, Yokohama, Japan

## Abstract

The purpose of the study was to compare the two mucin secretogogues, diquafosol (DQS) and rebamipide (RBM), for the treatment of dry eye syndrome (DES) in office workers. Dry eye patients using computers for >4 h/day were randomly assigned treatment with either DQS or RBM. Main outcomes measures included changes in tear film break-up time (TBUT) and subjective symptoms assessed by the Dry Eye-Related Quality of Life Score (DEQS). The subjects had scheduled examinations at 0 and 4 weeks, and the examinations at 2 and 8 weeks were optional. Changes in keratoconjunctival fluorescein score and a patient satisfaction questionnaire were also recorded. Both groups showed significant improvements in the DEQS scores at 2, 4, and 8 weeks following the initiation of the study. Both groups showed significant increases in the TBUT at 2 and 4 weeks. No significant difference was found between the DQS and RBM groups at any time periods. Patients reported more comfort with the use of DQS compared with the use of RBM. No local or systemic side effects were noted. The results of the present study indicated that both DQS and RBM were effective for the treatment of DES in office workers.

## Introduction

Dry eye syndrome (DES) is a common eye disorder with an increasing incidence that affects more than 10 million people in Japan^1^. A recent report by the Asia Dry Eye Society defines DES as follows: “Dry eye is a multifactorial disease characterized by unstable tear film causing a variety of symptoms and/or visual impairment, potentially accompanied by ocular surface damage”^2^. Although DES primarily affects females and the elderly, it is also a concern among young, active workers. A recent study reported that more than half of Japanese office workers suffered from DES, and the use of a computer for >4 h/day was a risk factor for DES^3^. In addition, studies have shown that DES is associated with general health problems and decreased productivity, including a decreased quality of sleep, depression, and impaired subjective happiness^4–13^.

DES has been primarily treated using artificial tears and antiinflammatory therapies^14–16^. Two kinds of mucin secretogogues, diquafosol sodium [3% Diquas^®^ ophthalmic solution (DQS), Santen Pharmaceutical, Osaka, Japan; chemical name, tetrasodium P1,P4-bis(5′-uridyl) tetraphosphate] and rebamipide [2% Mucosta^®^ ophthalmic suspension (RBM), Otsuka Pharmaceutical, Tokyo, Japan; chemical name, (2RS)-2-(4-chlorobenzoylamino)-3- (2-oxo-1,2-dihydroquinolin-4-yl) propanoic acid] have recently become commercially available in Japan. While both formulations increase tear film mucins, they have different mechanisms of action. DQS is a purinergic P2Y2 receptor agonist that increases mucin expression and its secretion from goblet cells in mice^17^, rats^18^, rabbits^19^, and humans^20^. RBM is a mucoprotective drug, and it has been used for the treatment of gastoric/duodenal ulcers in Japan. RBM has been shown to increase the production of mucins from corneal or conjunctival tissues either in cultured cells^21^ or in animal models^22,23^. Unlike DQS, RBM has been shown to increase the goblet cell numbers^24,25^. Uchino Y recently reported that RBM increased MUC 16 protein synthesis in human corneal epithelial cells^26^. In addition, RBM has been shown to have antiinflammatory effects^27–29^. Both drugs have been reported to be effective for various kinds of DES including Sjogren’s syndrome, non-Sjogren’s aqueous-deficient DES, and DES with tear film instability^30–41^. However, there has been no large-scale prospective report that compared the efficacy and safety of these drugs. In the present study, we conducted a prospective, randomized, clinical trial to determine the efficacy and safety of DQS and RBM for the treatment of DES in office workers that used computers.

## Results

### Participants

**Figure 1:**
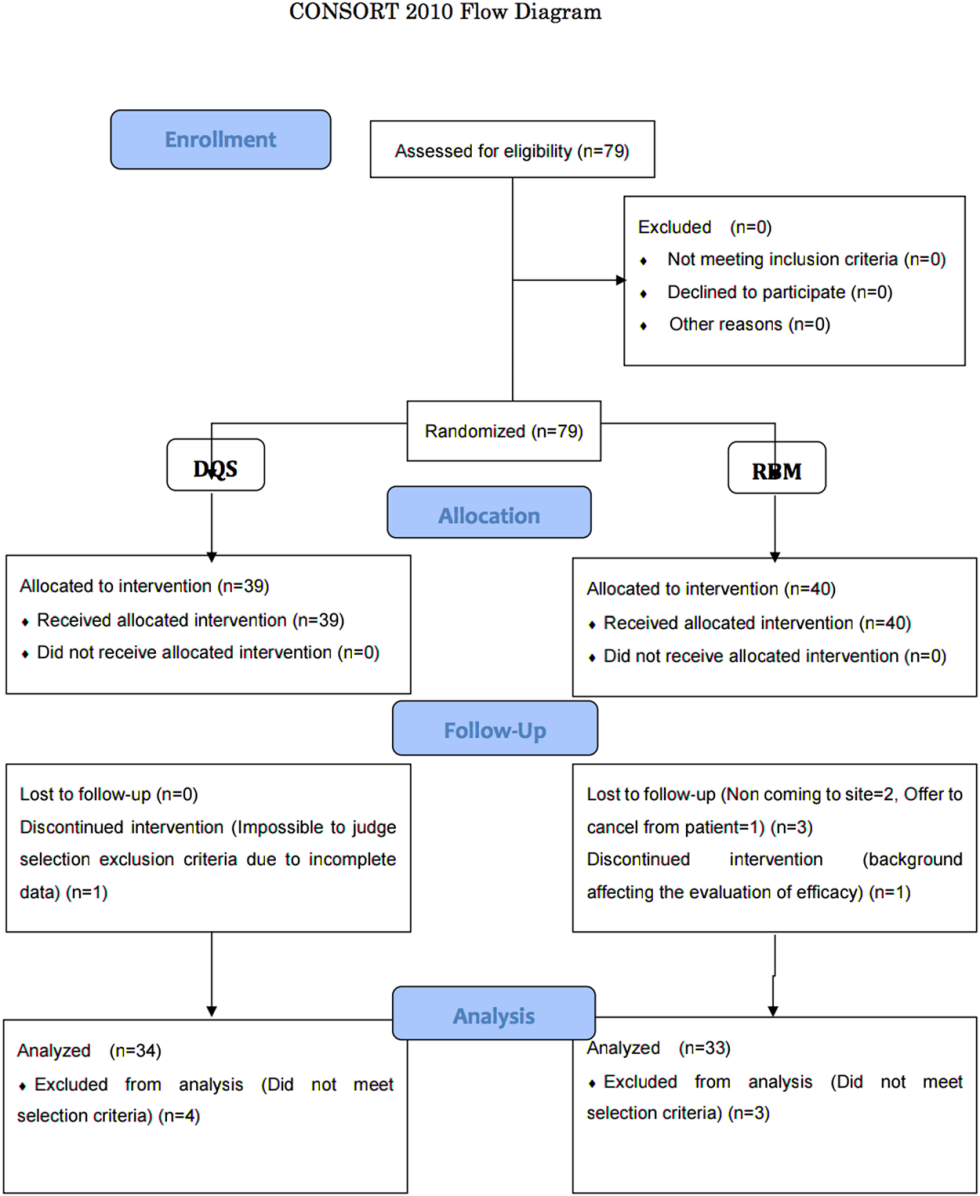
CONSORT (Consolidated Standards of Reporting Trials) 2010 flow diagram.

A diagram of participant flow is shown in Fig. 1. Of the 79 workers enrolled, 12 dropped out of the study due to the following: 7 workers did not come for the follow-up, 3 workers discontinued the study due to self-judgment, 1 worker moved to another place, and 1 worker required ophthalmic surgery (chalazion excision).

Table 1 shows a demographic profile of the workers in the study. There were 20 males and 47 females with a total mean age of 40.0 ± 10.2 years. Thirty-four and 33 eyes were allocated to the DQS and RBM groups, respectively. There was no significantly different parameter between the two groups (Table 1).

**Table 1:** Demographic profile of subjects.

		Diquafosol (n = 34)	Rebamipide (n = 33)	P value
Age (years, mean±SD)		38.2 ± 9.7	41.5 ± 9.6	0.17
Sex (male:female)		10:24	10:23	1.00
Mean computer use time	4–6 h/day	9	7	0.44
	6–8 h/day	10	8	
	>8 h/day	15	18	
Other eye Drops used	None	29	28	1.00
	Artificial tears	0	3	
	Hyaluronates	3	2	
	Others	2	0	
BSCVA		1.23 ± 0.15	1.18 ± 0.10	0.11
Mean TBUT (s)		3.45 ± 1.02	3.20 ± 0.84	0.28
Schirmer’s value (mm/5 min)		13.7 ± 9.50	15.2 ± 10.7	0.56
Mean QOL score		44.7 ± 20.2	48.6 ± 18.0	0.41

BSCVA, best spectacle-corrected visual acuity; TBUT, tear film beak-up time; QOL; quality of life (determined by the Dry Eye-Related Quality of Life Score test).

### Changes in subjective symptoms

**Figure 2:**
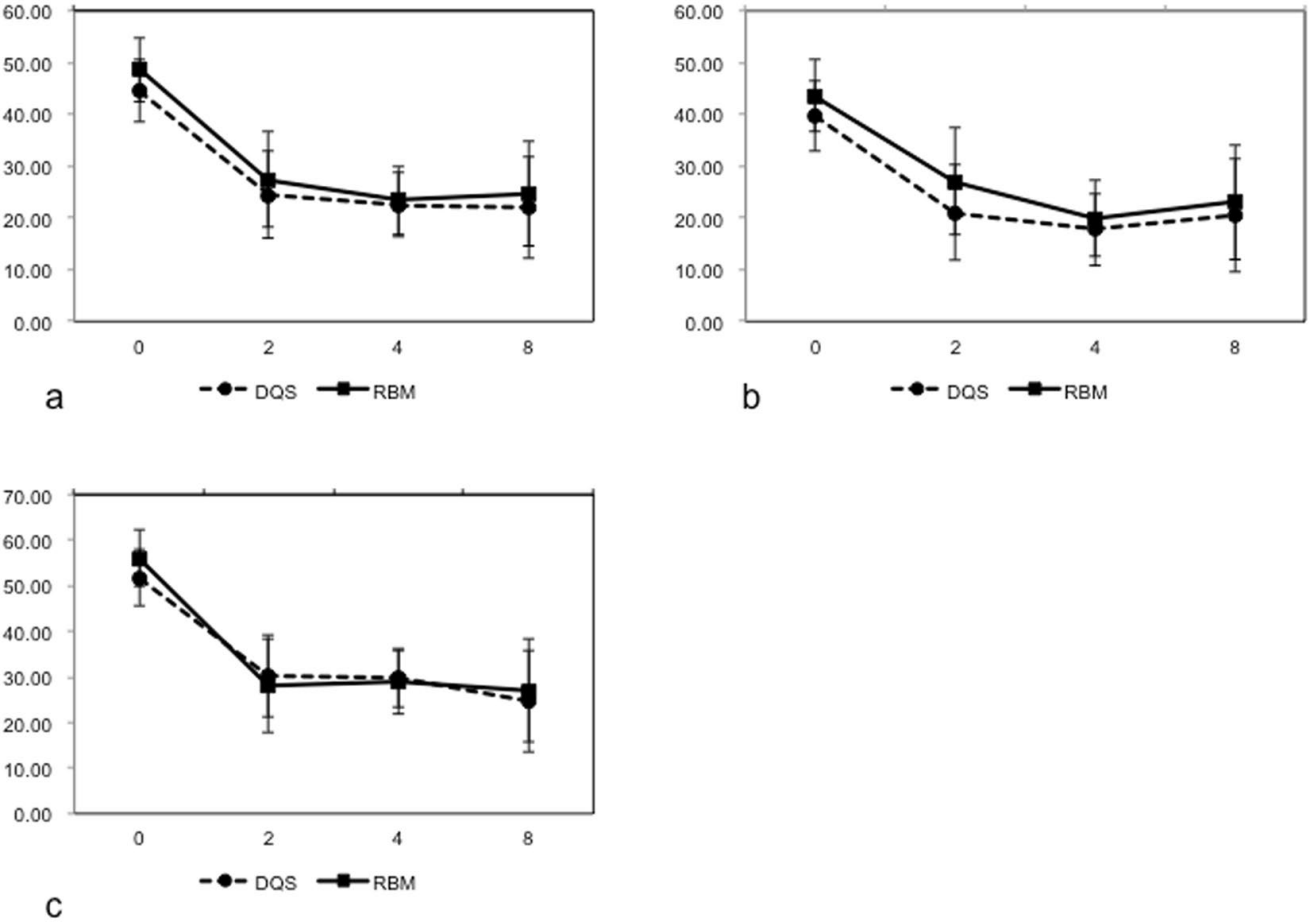
Changes in subjective symptoms in diquafosol and rebamipide groups assessed by the Dry Eye-Related Quality of Life Scores: (**a**) changes in the total score, (**b**) changes in the ophthalmic symptoms, and (**c**) changes in the quality of life-related scores.

Figure 2A shows the changes in total subjective symptoms of the DEQS and RBM groups. Both groups showed significant improvements in symptoms at 2, 4, and 8 weeks following the initial treatments (P < 0.0001 at all time points). No significant difference was noted between DQS and RBM groups throughout the observation period.

Figures 2B,C shows the changes in ocular symptoms and daily life-related symptoms. In ocular symptoms, both DQS and RBM groups showed significant improvements in symptoms at 2, 4, and 8 weeks following the initial treatments (P < 0.0001 at all time points). In daily life-related symptoms, both groups showed significant improvements in symptoms at 2 (P < 0.0001 and P = 0.0009 in the DQS and RBM groups, respectively), 4 (P < 0.0001 in both groups), and 8 weeks (P = 0.0004 and 0.0002 in the DQS and RBM groups, respectively) following the initial treatments. No significant difference was found between the two groups throughout the observation periods.

### Changes in the TBUT and fluorescein scores

**Figure 3:**
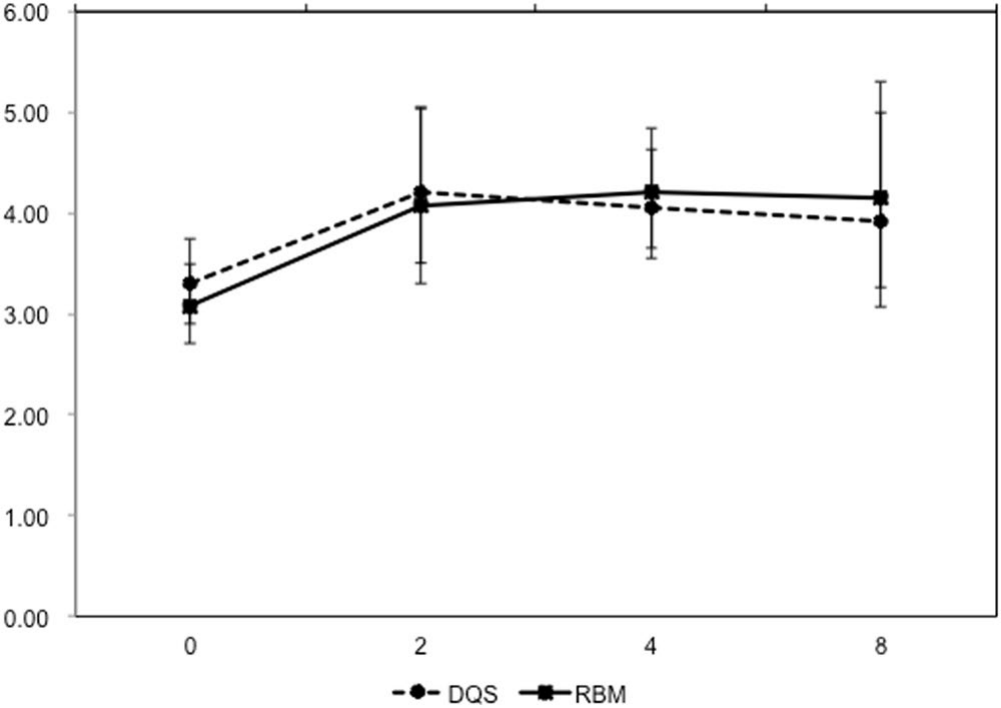
Changes in tear film break-up times.

Both the DQS and RBM groups showed significant prolongation of TBUT at 2 and 4 weeks following initiation of the treatment, compared with pretreatment values (Fig. 3). Only the RBM group showed a significant increase in the TBUT at week 8 (P = 0.022). No significant difference was noted between the DQS and RBM groups at any period.

**Figure 4:**
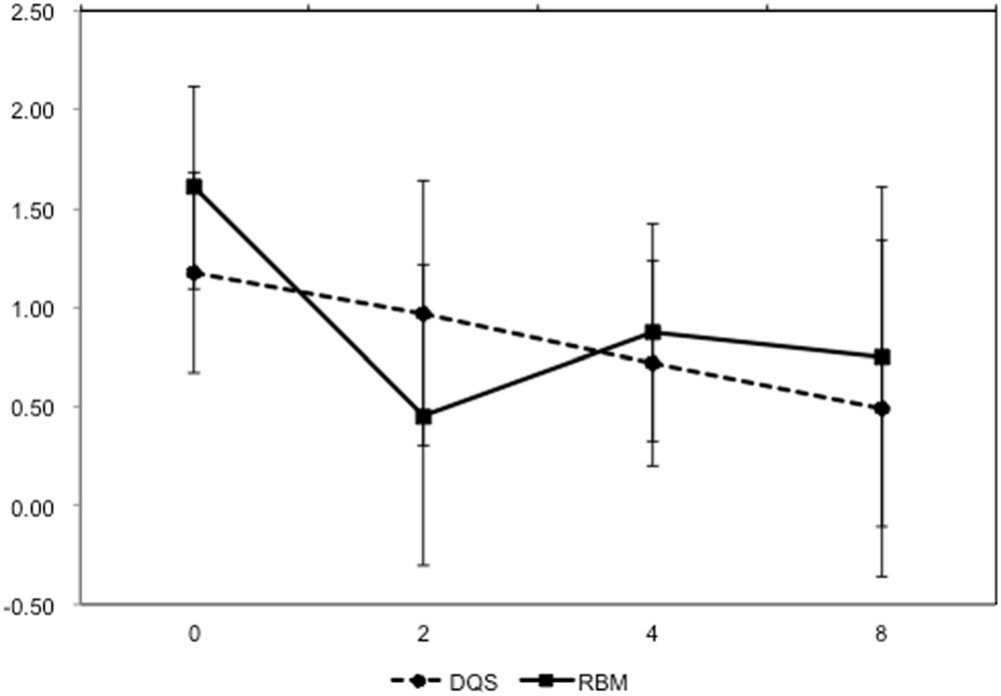
Changes in fluorescein staining scores.

The RBM group showed significant decreases in fluorescein scores at 2 (P = 0.0023), 4 (P = 0.0054), and 8 (P = 0.044) weeks, but not in the DQS groups compared with week 0. There was no significant difference between the DQS and RBM groups during any study period (Fig. 4). No local or systemic side effects were noted in both groups.

### Questionnaire

The results of the questionnaire are shown in Table 2. Twenty-eight and 23 responses were obtained from the DQS and RBM groups, respectively. The workers reported that comfort was better in the DQS group compared with the RBM group (P = 0.042). There was no significant difference between the two groups regarding “easy to use” and “willing to use more.”

**Table 2:** Patients’ preferences for diquafosal and rebamipide eye drops using the questionnaire.

Questionnaire	Diquafosol (n = 34)	Rebamipide (n = 33)	P value
Comfort			
Very good	5 (17.9%)	1 (4.3%)	0.042
Good	6 (21.4%)	5 (21.7%)	
Fair	16 (57.1%)	11 (47.8%)	
Bad	1 (3.6%)	6 (26.1%)	
Very bad	0 (0%)	0 (0%)	
Easy to use			
Very good	5 (17.9%)	1 (4.3%)	0.62
Good	10 (35.7%)	14 (60.9%)	
Fair	11 (39.3%)	5 (21.7%)	
Bad	2 (7.1%)	2 (8.7%)	
Very bad	0 (0%)	1 (4.3%)	
Willing to use more			
Very positive	3 (10.7%)	1 (4.3%)	0.20
Positive	13 (46.4%)	8 (34.8%)	
Fair	8 (28.6%)	9 (39.1%)	
Negative	4 (14.3%)	5 (21.7%)	
Very negative	0 (0%)	0 (0%)	

### Factor analyses

The results of the factor analyses are shown in Table 3. There were two factors (factors 3 and 4) that showed a relatively strong association with the use of eye drops. For factor 3, “dryness” needed improvement, usefulness (i.e., comfort, easy to use, willing to use more) was preferred in the DQS group, and “heavy feeling” needed improvement in the RBM group. The results of factor 4 showed that the epithelial damage responded to DQS treatment in workers with limited computer times, for users of other DES eye drops for more than 2 weeks, and for workers with low Schirmer’s test values. DEQS scores, the presence of photophobia, and worsening of DES symptoms while reading improved more in workers in the RBM group who had characteristics of the above workers.

**Table 3:** The results of the factor analyses.

Factors	Factor1	Factor2	Factor3	Factor4	Factor5
Drugs	−0.176	0.164	**0.439**	**−0.425**	0.339
Sex	−0.249	0.042	−0.162	0.025	**−0.718**
Age	−0.065	0.149	−0.035	−0.125	−0.089
Mean duration of computer work	−0.231	−0.091	−0.102	**−0.478**	−0.206
Use of other dry eye medications	0.029	0.196	−0.002	**0.414**	−0.311
Corrected visual acuity	0.069	0.080	−0.234	0.258	**0.703**
Schirmer’s I test	0.037	−0.061	0.228	**−0.583**	0.040
Comfort for eye drop use	−0.154	−0.017	**0.806**	−0.210	0.034
Easy to use	0.014	−0.296	**0.667**	−0.134	−0.206
Willing to use more	0.028	−0.211	**0.813**	0.057	−0.113
Δ log(BUT)	0.166	−0.214	−0.114	−0.027	**0.559**
Δ Staining scores	−0.113	−0.133	0.062	**−0.417**	−0.192
Δ DEQS score (total)	**0.921**	0.050	−0.061	**0.483**	0.179
Δ DEQS score (ocular symptom)	**0.922**	0.045	0.021	0.200	0.237
Δ DEQS score (QOL)	**0.753**	0.046	−0.119	0.638	0.098
Δ Foreign body sensation	**0.601**	0.221	0.000	−0.056	0.344
Δ Dry sensation	**0.574**	0.102	**0.388**	0.087	0.221
Δ Ocular pain	**0.819**	−0.110	−0.084	0.320	0.000
Δ Eye strain	**0.680**	−0.210	0.183	0.093	0.099
Δ Heavy feeling	**0.604**	0.141	**−0.375**	0.120	0.069
Δ Eye redness	0.232	**0.595**	−0.209	0.111	0.056
Δ Difficulty in eye open	**0.660**	0.269	−0.115	0.247	**0.504**
Δ Blurring	**0.388**	0.346	−0.157	0.209	0.111
Δ Photophobia	**0.639**	−0.211	−0.116	**0.531**	0.081
Δ Newspaper reading	**0.403**	−0.207	−0.120	**0.727**	−0.230
Δ TV watching	**0.602**	−0.068	−0.119	−0.190	−0.182

*Factors with strong association were shown in bold.

## Discussion

Office workers are exposed to numerous risk factors for DES. Several studies have reported that prolonged computer use is associated with the development of DES. A recent study also reported that sedentary behavior and the resulting metabolic syndrome were risk factors for DES^5,8^. Furthermore, many office workers use soft contact lenses and/or are exposed to air-conditioning, which are both considered to be risk factors for DES. DES effects have been shown to decrease the productivity of office workers^42^. In a recent clinical study that examined 672 Japanese office workers who used computers, 65 workers (11.6%) were diagnosed with DES and 303 workers (54.0%) were diagnosed with probable DES according to the Japanese Dry Eye Criteria^43^. Most of the subjects with probable DES had a decreased TBUT (<5 s), positive symptoms without measurable ocular surface epitheliopathy, and decreased tear secretion (i.e., a short TBUT-type DES). Decreased TBUT is an important parameter for DES in office workers, so this parameter was chosen as one of the main outcomes in the study. Overall, 44 eyes were categorized as short-BUT-type DES.

Ocular surface mucins have been implicated in the pathogenesis of DES. Uchino *et al*. reported that the MAC5AC concentration in tears was significantly lower in office workers that used computers for long periods of time compared with short-term computer users^44^. Both DQS and RBM are mucin secretogogues; thus, they are expected to be effective for the treatment of DES in office workers. Since it became available in Japan in 2011, DQS ophthalmic solution has been used and proven effective for treating various types of DES, including aqueous-deficient, short TBUT-type DES and postoperative DES following cataract or laser *in situ* keratomileusis^34,36,45–49^. Due to its unique properties for mucous tissue protection, RBM has been widely used for the treatment of gastric and duodenal ulcers. As eye drops, RBM was launched in the Japanese market in 2012 for the treatment of DES. It has been reported to increase the production of mucin-like substances in the cornea and conjunctiva, increase the number of goblet cells, suppress expression of cytokines, and attenuate tumor necrosis factor-α-induced barrier disruption in the corneal epithelium^21,23,28^. Although both DQS and RBM promote mucin secretion in tears, there have been many reports indicating their own unique mechanisms of action. DQS increases tear film quantity for up to 30 min following instillation in normal human subjects, which was much longer than artificial tears and hyaluronates^50^. Due to its mechanism of action, DQS is considered to be useful for aqueous-deficient DES. In addition, DQS upregulates the expression of secretory mucins (e.g., MAC5AC) and membrane-bound mucins (e.g., MUC1, MUC4, and MUC16) that are responsible for the wettability of the ocular surface epithelium. Recent studies also reported that P2Y2 receptors are expressed in meibomian glands; Arita *et al*. reported the potential usefulness of DQS for the treatment of obstructive meibomian gland dysfunction^51^. In contrast, RBM has been shown to be effective for various ocular surface diseases other than DES, including allergic conjunctivitis, filamentary keratitis, conjunctivochalasis, lid wiper epitheliopathy, and superior limbic keratoconjunctivitis^52–54^. The underlying mechanism is not totally clear; however, there are two major hypotheses that explain RBM`s unique properties. One is that RBM has anti-inflammatory and antioxidant properties^23^. The second mechanism is that RBM increases the number of goblet cells^24^. These two mechanisms are consistent with RBM being a potential treatment for ocular surface diseases related to persistent inflammation and friction between the ocular surface epithelium and lid margins. RBM also increased the expression of membrane-bound mucins *in vitro* ^55^.

The results of the present study indicated that both DQS and RBM were effective in alleviating DES symptoms and prolonging TBUT. The effects were found as early as 2 weeks. Factor analyses indicated that DQS was more effective in workers using computers for long periods of time or in eyes with decreased Schirmer’s test values. These findings are consistent with the mechanism of action of DQS, involving promotion of aqueous secretion from the conjunctival epithelium. In addition, recent studies reported that DQS is effective for contact lens users with DES^49,56^. Although contact lens users were excluded from the present study, these observations may expand the usefulness of DQS in office workers. RBM was also an effective treatment for eyes with ocular surface epithelial damage (Fig. 4). It is notable that some workers preferred using DQS rather than RBM. RBM is a single dose unit drug that is a white emulsion, so some patients may have had ambivalent feelings about using such an emulsion in their eye. Because DES patients need to apply eye drops over a long period, a comfortable eye drop generally may be selected to continue the therapy.

There were some limitations in the present study. First, as previously mentioned, we did not include contact lens wearers in the study, although many workers with DES in the office wear disposable soft contact lenses, so the efficacy of DQS and RBM for contact lens wearers should be investigated in the future. In addition, we did not study the long-term effects of the drugs. Because both drugs improved DES symptoms and signs when used over an extended period of time, continuous use of the drugs would further increase their efficacy^32,37^. Also, the present study did not elucidate the mechanism of action why DQS and RBM were effective for the DES in office workers as no cytological/biological studies including impression cytology or mucin measurements. Further studies are needed to determine the long-term effects.

In summary, in a prospective, randomized, clinical study, we found that DQS and RBM were both effective for alleviating irritating symptoms and prolonging the TBUT of DES in office workers. Because the drugs have different mechanisms of action and usability, they may be used differently based on the subtypes of DES and the patients’ preferences.

## Methods

### Study design and participants

This was an open-labeled, prospective, randomized, multicenter clinical trial involving one university hospital and six private clinics in the Tokyo metropolitan area. The protocol adhered to the principles of the Declaration of Helsinki. Written informed consent were obtained by all subjects after explaining the purpose and potential risks of the study. The study was approved by the internal review board of each participating hospital/clinic, and the clinical trial was registered in the Clinical Trial Registry of the University Hospital Medical Information Network (UMIN-CTR; UMIN000012742) with the date of registration (01/01/2014). The study was approved by the internal review board of the Tokyo Dental College (I-13-12). The study was also approved by the internal review board of the IRB in the Keishokai Medical Corporation that manages Ryogoku Eye Clinic, Shinjuku Eye Clinic, and Iidabashi Eye Clinic in October 2013. Other participating clinics including Shimazaki Eye Clinic, Ichikawa Shapo Eye Clinic, and Smile Eye Clinic charged the IRB of the Keishokai Medical Cooperation with the deliberation of the ethical validity of the study protocol.

Inclusion criterion included: 1) DES or suspected DES according to the criteria of the Japan Dry Eye Society^57^, 2) in the Dry Eye-Related Quality of Life Score (DEQS), a general condition score >4 and any of the scores regarding bothersome ocular symptoms >3, 3) full-time office workers who used computers an average of >4 h/day, and 4) workers 20–60 years of age. The following workers were excluded: users of either DQS or RBM within 2 weeks of the study initiation, users of systemic medications that can influence tear/ocular surface conditions, pregnant females, workers with active ocular surface disorders other than DES, workers with abnormal eyelids or blinking, workers with a history of ocular surgeries, workers using punctual plugs, and contact lens wearers. There were 5 subjects in each group who have been using artificial tears at the time of initiation of the study, and they were instructed to continue using the eye drops throughout the study period without changing the frequency.

The diagnostic criteria of the Japan Dry Eye Society are: 1) positive symptoms, 2) qualitative or quantitative disturbance of the tear film (Schirmer I test equal or less than 5 mm/5 min or BUT equal or less than 5 sec), 3) keratoconjunctival epithelial damage (staining score greater than 3 points). The presence of all criteria renders a diagnosis of definite dry eye and the presence of two out of the three criteria renders a diagnosis of probable dry eye^57^. In the present study, 3 and 10 eyes in the DQS and RBM groups, respectively, were “definite dry eye”, while 31 and 23 eyes in the DQS and RBM groups, respectively, were “probable dry eye”. Vast majority of the eyes with “probable dry eye” had positive symptoms and BUT with equal or less than 5 seconds, while keratoconjunctival staining scores with less than 3 points (30 and 23 eyes in the DQS and RBM groups, respectively). The workers were randomly divided into two groups using an envelope method, with use of DQS eye drops six times/day or RBM eye drops four times/day. If workers had been using other eye drops, they continued to use them during the study period at the same frequency. The workers had scheduled examinations at 0 and 4 weeks after the initiation of treatments. Examinations at 2 and 8 weeks were optional.

Previous studies reported that DQS or RBM improved subjective symptom scores 30–60% in DES patients^33,36^. We assumed that clinically meaningful changes in symptoms were 20%, with a 30% standard deviation. A sample size of 40 eyes (80 eyes in total) in each group was chosen to provide at least 80% power to detect a 20% difference in rejection rates using a one-sided α-error level of 0.05.

### Examinations

The primary outcome of the study included changes in subjective symptoms assessed by the DEQS, and changes in tear film break-up time (TBUT). The DEQS is a validated questionnaire consisting of 15 items and two subscales involving the Impact on Daily Life and Bothersome Ocular Symptoms^58^; six items in the questionnaire pertain to bothersome ocular symptoms and nine items consider impacts on daily life. All patients first answered the frequency of symptoms and disability, and then answered the degree of each item. The calculated summary score ranged from 0–100, with a higher score representing a greater disability. In addition to the DEQS questionnaire, the patients’ preferences for drug treatment were examined in a different set of questionnaires consisting of three questions: 1) Were the eye drops comfortable? 2) Were the eye drops easy to use? 3) Are you willing to continue using the eye drops? The answer to each question was graded on a scale using five degrees.

The TBUT was chosen as the main outcome because impaired tear film stability is considered to be the major mechanism that causes dry eye and/or visual impairment^2^. The TBUT was measured after instillation of a minimum amount of preservative-free 1% fluorescein dye. The time until the first dry spot appeared was recorded, and the average of three measurements was used in subsequent analyses. The second and third outcome measures included the patients’ preference using the questionnaire and the ocular surface damage assessed by fluorescein staining scores. Staining scores were evaluated using van Bijsterveld scoring in which the staining intensity was measured semiquantitatively using a range from 0–3 in the cornea and nasal/temporal bulbar conjunctiva (a maximum of 9 points)^59^. To evaluate secretion of tears, the standard Schirmer’s test without topical anesthesia was performed more than 30 min after determining the TBUT or the vital stain scores to avoid affecting the two tests. Cutoff values were determined according to the Japanese Dry Eye Criteria. Local or systemic side effects were also recorded.

### Data collection and statistical analysis

Clinical data were sent to the data center, and the eligibility of the subjects for this study were checked by a masked examiner. Continuous background variables were summarized using the mean and standard deviations, and the *t*-test was used for a comparison between treatment groups. Categorical background variables were summarized using count and percentages, and Fisher’s exact test and the Cochran–Mantel–Haenszel test were used for their comparisons. To evaluate changes in outcome variables and to compare treatment groups in treated eyes, mixed effects models were used with each time point, treatment, and interaction as fixed effects, with the patients as a random effect.

To describe the structural relationships among the treatment, background variables, and outcomes, factor analyses were used with the principal component method and Promax rotation. Fifty-four patients and 28 variables, including sex, age, mean computer work time, other eye drops for DES, visual acuity, Schirmer’s test as a background variable, questionnaire results, and changes in DEQS as outcomes were used in the analyses.
